# Temporal Trends and Predictors of Mortality in Heart Failure with Reduced Ejection Fraction: The Role of Renal Function and Left Ventricular Improvement

**DOI:** 10.3390/jcm15166223

**Published:** 2026-08-11

**Authors:** Andrea Lopez-Lopez, Estefanía Rivas Vázquez, Margarita Regueiro-Abel, Carmen Cristina Álvarez-Suárez, Charigan Abou Johk-Casas, Juliana Elices-Teja, Ramón Ríos Vázquez, Germán Santamarina-Pernas, Carlos González-Juanatey

**Affiliations:** 1 Department of Cardiology, Hospital Universitario Lucus Augusti, 27002 Lugo, Spaincarlos.gonzalez.juanatey@sergas.es (C.G.-J.); 2 CardioHULA Research Group, Instituto de Investigacion Sanitaria de Santiago de Compostela IDIS, 27002 Lugo, Spain; estefania.rivas.vazquez@sergas.es; 3 Doctorate Program, Department of Anatomy, Animal Production and Veterinary Clinical Sciences, Universidad de Santiago de Compostela, 27001 Lugo, Spain; 4 Faculty of Veterinary Medicine, Universidad de Santiago de Compostela, 27001 Lugo, Spain; 5 Nursing School, Universidad de Santiago de Compostela, Campus Lugo, 27001 Lugo, Spain

**Keywords:** heart failure, reduced ejection fraction, left ventricular recovery, renal function, mortality, risk stratification, cardiovascular death, non-cardiovascular death

## Abstract

**Background/Objectives**: Traditionally, risk stratification in heart failure with reduced ejection fraction (HFrEF) patients has been a static process. This study aims to identify clinical predictors and causes of mortality at different stages of the disease, distinguishing between early mortality (≤1 year) and late mortality (>1 year), as well as cardiovascular and non-cardiovascular causes. **Methods**: This is an observational, longitudinal study of a cohort of 1510 patients with HFrEF followed up in a specialist heart failure unit between 2011 and 2023. Patients were categorized into three groups: alive (*n* = 1093), early mortality (*n* = 55) and late mortality (*n* = 362). Multivariate Cox regression models were used to identify mortality independent predictors in each period. **Results**: During the first year, cardiovascular and non-cardiovascular mortality showed a relatively balanced distribution, with a slight predominance of non-cardiovascular causes (52.8%). Progression of heart failure remained the leading individual cause of early death (38.2%). Independent predictors of early cardiovascular death included ischemic etiology (HR 2.78; 95% CI: 1.20–6.40; *p* = 0.01), NYHA functional class (HR 4.38; 95% CI: 2.18–8.81; *p* < 0.001) and renal function (HR 0.60; 95% CI: 0.44–0.84; *p* = 0.002). In contrast, late mortality (>1 year) was predominantly due to non-cardiovascular causes (59.7%), notably cancer (24.3%) and infections (7.5%). Late all-cause mortality was independently associated with age (HR 1.35; 95% CI: 1.17–1.56; *p* < 0.001), hypertension (HR 1.38; 95% CI: 1.06–1.81; *p* = 0.015) and ischemic etiology (HR 1.45; 95% CI: 1.16–1.80; *p* < 0.001). In the follow-up, the improvement of left ventricular ejection fraction (LVEF) emerged as a protective factor against long-term all-cause mortality (HR 0.77; 95% CI: 0.63–0.94; *p* = 0.01) and, even more markedly, against late cardiovascular mortality (HR 0.47; 95% CI: 0.33–0.66; *p* < 0.001), whereas baseline LVEF at diagnosis lacked significant predictive value in either model. **Conclusions**: Mortality in HFrEF is a dynamic process. Early survival is determined by hemodynamic stability, renal function and functional severity, whilst late survival is predominantly driven by non-cardiovascular comorbidities, biological aging, and ventricular remodeling trajectory. The improvement of LVEF during follow-up is a more reliable prognostic marker than the baseline measurement, suggesting that clinical management should shift from early pharmacological optimization towards a multidisciplinary approach focused on long-term non-cardiovascular risk.

## 1. Introduction

Heart failure (HF) remains a major challenge for global public health, affecting more than 64 million people worldwide [1,2]. This condition is one of the leading causes of morbidity and mortality globally, placing a heavy clinical and healthcare burden on health systems [1]. According to the contemporary European Society of Cardiology Heart Failure III Registry (ESC HF III), approximately 58% of all patients with HF are classified as having heart failure with reduced ejection fraction (HFrEF), representing the most prevalent phenotype across European centers [3].

Despite therapeutic advances that have transformed the prognosis of the disease in recent decades, heart failure with reduced ejection fraction (HFrEF) remains associated with significantly high morbidity and mortality [4]. Patients with HFrEF are at high risk of adverse events and mortality, a vulnerability that is particularly pronounced in the first few years following diagnosis.

The course of patients with HFrEF is not a linear process. A critical period known as the vulnerability phase is recognized, spanning the first few months following an initial diagnosis or acute decompensation. During the first year, the risk of readmission and death is highest, frequently determined by hemodynamic severity and the persistence of congestion [3,5,6].

Traditionally, risk stratification in heart failure has been based on prognostic models that estimate overall mortality, such as the Seattle Heart Failure Model [7]. However, these models assess risk statically and often fail to capture the dynamic nature of the disease, the temporal evolution of the mortality profile, or the functional recovery of left ventricular function. In this context, recent studies suggest that mortality in patients with HF is not homogeneous, but may vary over time, with significant changes in the relative contribution of cardiovascular and non-cardiovascular causes [3,4,8,9].

An emerging phenomenon in recent decades is the change in the mortality profile of these patients. Whilst advances in the four pillars of guideline-directed medical therapy (GDMT)—beta-blockers, neprilysin and angiotensin receptor blockers, sodium-glucose cotransporter 2 inhibitors and aldosterone receptor antagonists—have succeeded in drastically reducing deaths due to progression of HF and sudden death, the burden of non-cardiovascular comorbidities has taken on renewed significance [10,11]. This high burden of concomitant conditions plays a significant role in disease progression and prognosis, particularly in more advanced stages, contributing to a more complex and systemic clinical profile [6]. In fact, this early period of titration represents a key window for rapid optimization of GDMT, as demonstrated in the STRONG-HF trial [12]; however, clinical instability and comorbidities such as renal impairment frequently hinder treatment titration [13].

In patients undergoing long-term follow-up, factors such as renal dysfunction, diabetes mellitus and cancer emerge as critical determinants of long-term prognosis [14]. In a systematic review of phase III HFrEF trials, cancer emerged as a leading non-cardiovascular cause of death, remaining stable over time while cardiovascular mortality declined [15]. Similarly, in a Spanish HF clinic cohort, non-cardiovascular deaths rose progressively over two decades, with cancer and infections gaining increasing relevance [16]. Consistent with these observations, in the ESC-HFA EORP Heart Failure Long-Term Registry, post-discharge mortality increased in a graded fashion with the number of non-cardiac comorbidities. Notably, chronic kidney disease, anemia, chronic obstructive pulmonary disease, diabetes mellitus, peripheral artery disease, prior stroke, and depression were independently associated with an increased risk of post-discharge mortality, with the latter three demonstrating a prognostic impact specific to the HFrEF subgroup [17]. Collectively, these findings support a paradigm shift in which HFrEF increasingly behaves not as an isolated cardiac condition but as a systemic syndrome, where late mortality appears linked to biological aging and multimorbidity rather than to HF itself [5,18].

For all these reasons, there is a clinical need to differentiate mortality predictors according to the stage of the disease. Distinguishing between early mortality (≤1 year) and late mortality (>1 year), as well as between cardiovascular and non-cardiovascular causes, provides key information for better understanding the clinical course of patients with HFrEF and improving prognostic stratification through a more dynamic approach.

### Objectives

The main objective of this study was to identify the clinical characteristics, treatment profile and predictors of mortality in a cohort of patients with HFrEF. To this end, a chronological distinction was made between early mortality (≤1 year) and late mortality (>1 year) following the initial contact with a HF specialist unit.

Furthermore, the study aimed to analyze cardiovascular and non-cardiovascular mortality independently in both periods. This approach allows for the assessment of possible differences in prognostic determinants and risk factors according to the stage of disease progression.

## 2. Methods

### 2.1. Study Design and Patients

A single-center, retrospective, longitudinal observational study was conducted in a cohort of patients with HFrEF followed up at the HF Unit of the Cardiology Department in Lugo, Spain. A total of 1510 patients were consecutively included who commenced follow-up at the clinic between January 2011 and December 2023 inclusive, thus ensuring a minimum follow-up period of 2 years for the sample analyzed.

The baseline and index date for each patient was defined as the date of their first consultation at the specialist Heart Failure unit. Patients were referred to the unit through three primary clinical pathways, which included post-hospitalization discharge, direct referral from other outpatient clinics following an initial diagnosis, or immediate referral following an emergency department visit for acute decompensation. All clinical, laboratory, and echocardiographic variables analyzed as baseline characteristics were collected during this initial specialist visit.

Patients were eligible for inclusion if they were aged 18 years or older and had a documented diagnosis of heart failure with reduced ejection fraction, strictly defined by a left ventricular ejection fraction of 40% or less at the index baseline visit. Patients who had already demonstrated a recovered ejection fraction above 40% at the time of entry were excluded from the study. To ensure a representative real-world cohort, we included patients with both stable chronic heart failure and those recently stabilized after an acute decompensation or an emergency department visit. No exclusions were made based on heart failure etiology, meaning that individuals with ischemic cardiomyopathy and severe valvular disease were fully eligible. Furthermore, patients with significant comorbidities, such as active cancer, advanced chronic kidney disease, and those on maintenance dialysis, were included in the cohort. Patients with missing baseline clinical or echocardiographic data essential to verify the entry criteria were excluded from the final analysis, while missing follow-up data were handled using a complete-case approach.

The sample was divided into three groups according to clinical outcome: living patients (*n* = 1093), those who died during the first year (*n* = 55) and those who died after the first year of follow-up (*n* = 362). The mean total follow-up was 4.9 ± 3.5 years. In the early death group, it was 0.56 ± 0.26 years (203 ± 97 days) and in the late death group, 5.3 ± 2.99 years (1948 ± 1090 days).

### 2.2. Data Collection and Definitions

Clinical, demographic, laboratory and echocardiographic data, details of drug treatment and mortality records were obtained by reviewing patients’ electronic medical records. Relevant variables were collected, including age, sex, cardiovascular risk factors, the etiology of HF and renal function assessed by glomerular filtration rate.

Left ventricular ejection fraction (LVEF) was assessed by echocardiography (IE-33 and EPIQ-5, Philips, Bothell, WA, USA) at the start of follow-up and at subsequent check-ups to determine functional progression through the following. Patient management was carried out at each visit in accordance with the current clinical practice guidelines of the European Society of Cardiology (ESC) [19]. All patients underwent systematic titration and optimization of guideline-directed medical therapy during follow-up, according to the recommendations applicable at the time of treatment.

For the analysis, patients were classified according to their vital status and the time of death into three groups: living patients, patients who died during the first year of follow-up (≤1 year) after the initial consultation, and patients who died after the first year (>1 year) of follow-up. This classification allowed for a differentiated assessment of early and late mortality. The primary outcome was all-cause mortality, analyzed across two time periods: early mortality (≤1 year) and late mortality (>1 year).

Mortality events were adjudicated and validated by cardiologist investigators through systematic review of electronic medical records, hospital discharge summaries, emergency department logs and primary care documentation. Causes of death were classified using standardized clinical criteria. Cardiovascular mortality was defined as death due to heart failure progression, sudden cardiac death, acute coronary syndrome, or stroke. Non-cardiovascular mortality included deaths resulting from malignant neoplasms, infections or sepsis, severe functional decline, or other non-cardiac causes. In cases where the exact cause could not be definitively ascertained due to incomplete documentation, deaths were classified as unknown.

Ischemic etiology was defined based on a documented history of myocardial infarction, prior coronary revascularization (percutaneous coronary intervention or coronary artery bypass surgery), angiographically proven significant coronary artery disease (50% stenosis in at least one major epicardial vessel on invasive or computed tomography coronary angiography), or objective non-invasive evidence of inducible myocardial ischemia.

Respiratory comorbidities were defined as documented chronic lower respiratory diseases, including chronic obstructive pulmonary disease (COPD), asthma, and obstructive sleep apnea syndrome.

### 2.3. Statistical Analysis

Quantitative variables were expressed as mean ± standard deviation and qualitative variables as frequencies and percentages. Comparisons between groups were performed using the chi-square test or Fisher’s exact test for qualitative variables, and using ANOVA, Student’s *t*-test or non-parametric tests for quantitative variables, as appropriate.

An initial descriptive analysis was performed comparing the three defined groups. Subsequently, clinically relevant candidate predictors were prespecified a priori based on previous evidence as prognostic factors in patients with heart failure with reduced ejection fraction. These included age, sex, ischemic etiology, New York Heart Association (NYHA) functional class, estimated glomerular filtration rate (eGFR), baseline left ventricular ejection fraction (LVEF), diabetes mellitus, hypertension, baseline loop diuretic use and year of diagnosis. All prespecified candidate predictors were evaluated in univariable Cox regression analyses. Variables with a statistically significant association in the univariable analyses (*p* < 0.05) were entered into the initial multivariable Cox regression model. To obtain a parsimonious final model, variables were then removed sequentially in a manual stepwise manner until only statistically significant predictors remained. Given the limited number of events, particularly in the early mortality analyses, a parsimonious modeling strategy was adopted to reduce the risk of overfitting. Calendar year of diagnosis was handled according to the same variable-selection strategy as the remaining candidate predictors and was not forced into the multivariable models. Multicollinearity among candidate predictors was assessed using variance inflation factors (VIF), and no evidence of relevant multicollinearity was identified. Cardiovascular and non-cardiovascular deaths were considered mutually exclusive competing events. In models of cardiovascular mortality, non-cardiovascular deaths were censored at the time of occurrence; conversely, cardiovascular deaths were censored in models of non-cardiovascular mortality. Estimates are reported as cause-specific hazard ratios with 95% confidence intervals. Cumulative incidence functions were additionally estimated to describe the absolute incidence of each cause of death while accounting for the competing event. For analyses involving follow-up LVEF, only patients alive at one year with an available follow-up echocardiogram were included. Survival time was recalculated from the date of the follow-up echocardiographic examination until cardiovascular death or censoring.

The statistical analyses were performed using the IBM SPSS version 19.0 package (IBM, Armonk, NY, USA), considering a statistical significance level of *p* < 0.05. The proportional hazards assumption was formally assessed for all final Cox proportional hazards models using tests based on scaled Schoenfeld residuals, implemented with the cox.zph function from the R survival package (version 4.6-0). Both covariate-specific and global tests were examined. A two-sided *p*-value < 0.05 was considered evidence of non-proportional hazards.

### 2.4. Ethical Particulars

This study was approved by the Comité de Ética de la Investigación con Medicamentos de Galicia (CEIm-G) under the Registration Code 2025/378 in October 2025, prior to data extraction and analysis. Due to the retrospective, observational nature of this study using routine healthcare databases from the Hospital Universitario Lucus Augusti representing patient follow-up between 2011 and 2023, the requirement for individual written informed consent was officially waived by the ethics committee. All data were fully anonymized prior to analysis to ensure patient confidentiality in compliance with local data protection regulations.

## 3. Results

### 3.1. Cohort Description

The study included a total of 1510 patients diagnosed with HFrEF who were followed up at a specialist HF unit between 2011 and 2023 inclusive. Patients were divided into three groups according to their vital status and time of death: living patients (*n* = 1093), patients who died within the first year following the initial consultation (*n* = 55) and patients who died after the first year of follow-up (*n* = 362).

Significant differences were observed between the groups in terms of age and baseline renal function (Table 1). Patients who died, both within the first year and thereafter, were older and had a lower glomerular filtration rate (eGFR) compared with living patients. Although males predominated in the entire sample, their proportion was significantly higher in the groups of deceased patients. With regard to cardiovascular risk factors, the prevalence of hypertension and diabetes mellitus was notably higher in patients who died, with a higher frequency of diabetes being particularly evident in those with early mortality (Table 1).

The LVEF at the time of diagnosis did not show any statistically significant differences between the three groups analyzed. However, ischemic etiology was more prevalent in the sample, with a significantly higher percentage among deceased patients. Idiopathic and arrhythmic causes were the most prevalent in living patients. The remaining etiologies were grouped into a category termed ‘others’, which accounted for 14.5% of the total sample. Deceased patients showed higher respiratory comorbidities, cancer and atrial fibrillation than alive patients. LVEF at follow-up was calculated using LVEF values from the 2nd echocardiography performed on the patient after the 1st consultation at the unit, with a mean time of 365.2 ± 406.53 days (therefore, patients from the “death within 1 year” group do not have this data available). Patients who died after the 1st year showed lower FEVI values at follow-up than alive patients (*p* < 0.001) (Table 1).

The mean follow-up for the cohort was 1798 days (4.9 years) ± 1308 (3.5 years).

### 3.2. Baseline Pharmacological Treatment and Therapeutic Profile

Baseline medical treatment showed significant variations between the groups across almost all drug classes, with the exception of beta-blockers and anticoagulants. The use of beta-blockers was consistently high across the sample, standing at 96.6% overall (96.2% in living patients, 96.4% in those who died early, and 97.5% in those who died after the first year; *p* = 0.52) (Table 2).

A significant difference was observed in the use of the four therapeutic pillars in heart failure between the three groups. On the one hand, the use of renin–angiotensin–aldosterone system inhibitors (RAASIs), which include ACE inhibitors, ARBs and ARNIs, was significantly lower in patients who died early (90.9%) compared with living patients (97.3%) and those who died later (96.7%; *p* = 0.003). The prescription of loop diuretics was significantly higher in both groups of deceased patients (70.9% and 71.5%, respectively) than in those who survived (50.2%; *p* < 0.001), which may reflect a greater degree of baseline congestion in these patients (Table 2).

Furthermore, the use of SGLT2 inhibitors (SGLT2i) was significantly higher in the group of surviving patients (36.4%) compared with those who died after the first year (2.8%) and those who died early (20%; *p* < 0.001). A lower percentage of deceased patients received mineralocorticoid receptor antagonists (MRAs) than survivors (*p* < 0.001). Similarly, a lower rate of statin prescription was observed in deceased patients (38.1%) compared with those still alive (59.4%; *p* < 0.001) (Table 2).

This distribution of baseline pharmacological treatment suggests that patients with a poorer prognosis had a therapeutic profile consistent with greater clinical severity, characterized by increased use of loop diuretics and lower prescription rates for prognosis-modifying therapies.

### 3.3. Early Mortality (≤1 Year)

During the first year of follow-up following the initial consultation, 55 patients died. The mean follow-up period for this group was 202 ± 97 days. During this period, cardiovascular mortality accounted for 47.2% of deaths, whilst non-cardiovascular causes accounted for the remaining 52.8% (Table 3). Despite this balanced distribution, progression of heart failure remained the most frequent single cause of early mortality, accounting for 38.2% of all deaths.

**Figure 1:**
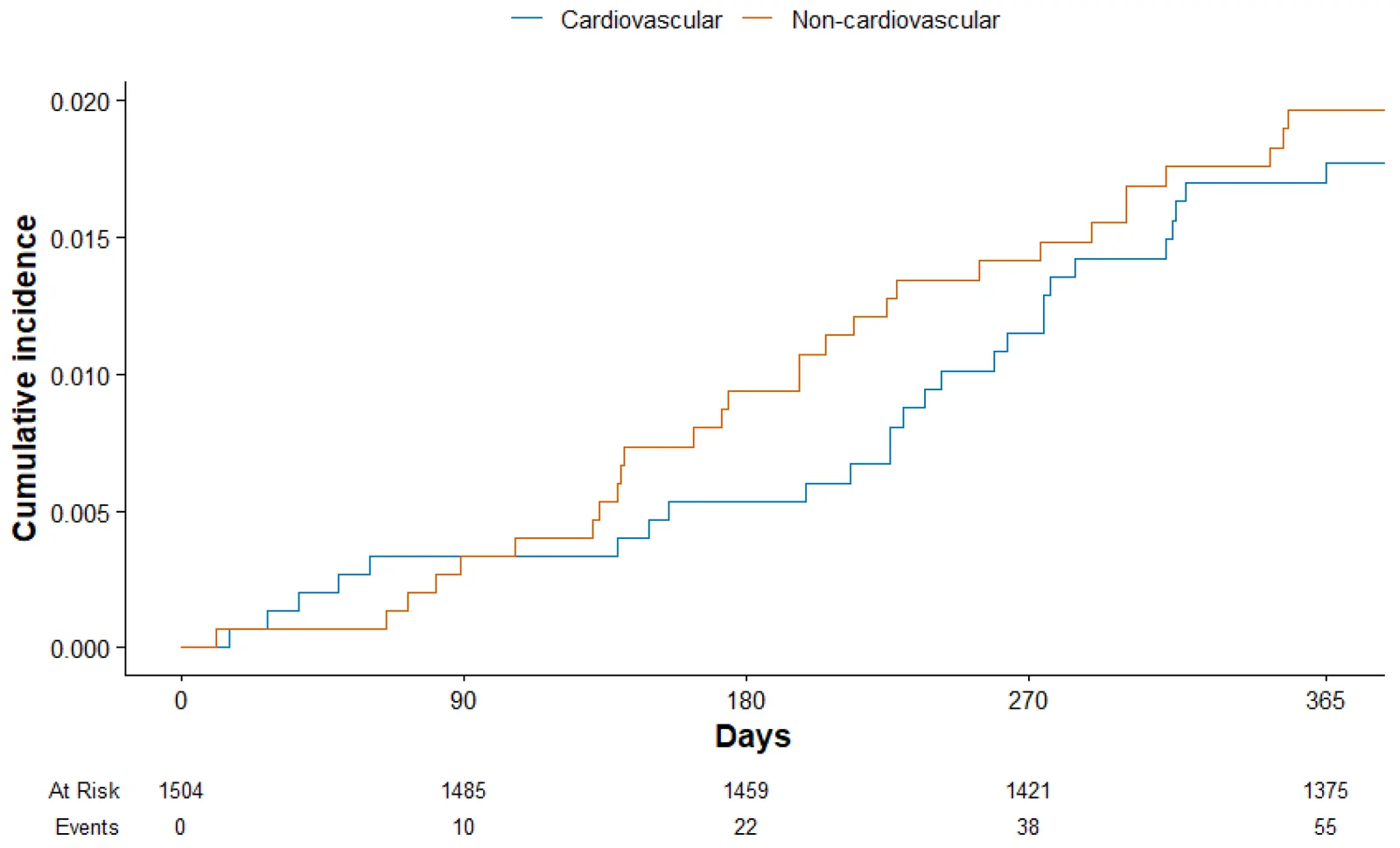
Cumulative incidence of cardiovascular and non-cardiovascular mortality during the first year of follow-up. The curves represent the cumulative incidence functions for cardiovascular mortality (blue line) and non-cardiovascular mortality (orange line) in the initial cohort (*n* = 1510) and had data from follow-up echocardiograms available. The lower section indicates the number of patients at risk and the cumulative number of events in each time interval.

The cumulative incidence curves show a distinct pattern throughout the first year of follow-up (Figure 1). During the first 90 days, the incidence of cardiovascular and non-cardiovascular mortality remained similar, with a slight divergence in favor of cardiovascular causes. However, from the first quarter until the end of follow-up, the trend in non-cardiovascular mortality showed a steady increase, consistently exceeding that of cardiovascular causes.

In the multivariate Cox analysis (parsimonious model) (Table 4), the factors independently associated with an increased risk of mortality within one year were ischemic etiology (HR 1.78; 95% CI: 1.03–3.08; *p* = 0.038), glomerular filtration rate (every 10 mL/min/1.73 m^2^; HR 0.71; 95% CI: 0.58–0.87; *p* = 0.001) and NYHA functional class (I, II or III/IV; HR 3.38; 95% CI: 2.07–5.52; *p* < 0.001).

#### 3.3.1. Premature Mortality from Cardiovascular Causes

The leading cause of early cardiovascular death was the progression of HF, accounting for 38.2% of all deaths in this group (Table 3). Other causes included sudden death (7.3%) and stroke (1.8%).

In the cause-specific multivariate Cox analysis, the factors independently associated with an increased risk of cardiovascular mortality within one year were ischemic etiology (HR 2.78; 95% CI: 1.20–6.40; *p* = 0.01), NYHA functional class (I, II or III/IV; HR 4.38; 95% CI: 2.18–8.81; *p* < 0.001) and renal function (every 20 mL/min/1.73 m^2^); HR 0.60; 95% CI: 0.44–0.84; *p* = 0.002) (Table 5).

#### 3.3.2. Early Mortality from Non-Cardiovascular Causes

Non-cardiovascular deaths during the first year totaled 29 cases. The predominant cause within this category was cancer, which accounted for 29.1% of total mortality during this period. Other causes included infections or sepsis (7.3%) and unknown causes (9.1%).

Unlike for cardiovascular mortality, the multivariate analysis (Table 6) did not identify any clinically or therapeutically significant variables that were independently associated with death from non-cardiovascular causes within the first year besides the NYHA value.

### 3.4. Late Mortality (>1 Year)

For the analysis of long-term mortality, deaths occurring during the first year were excluded, comparing the group of living patients (*n* = 1093) with those who died after one year of follow-up (*n* = 362). The mean follow-up in this group of deceased patients was 1948 ± 1090 days (5.3 ± 2.9 years). During this period, a shift in the mortality pattern was observed, with non-cardiovascular causes predominating, accounting for 59.7% of events, whilst cardiovascular causes fell to 40.3% (Table 7).

Despite this global trend reversal, the progression of HF remained the most common specific and individual cause of death across the entire cohort (28.5%). Sudden death was the second most common cardiovascular cause (10.5%). Within the spectrum of non-cardiovascular mortality, malignant neoplasms constituted the most relevant etiology (24.3%), followed by causes of unknown origin (11%) and infection or septic processes (7.5%) (Table 6).

**Figure 2:**
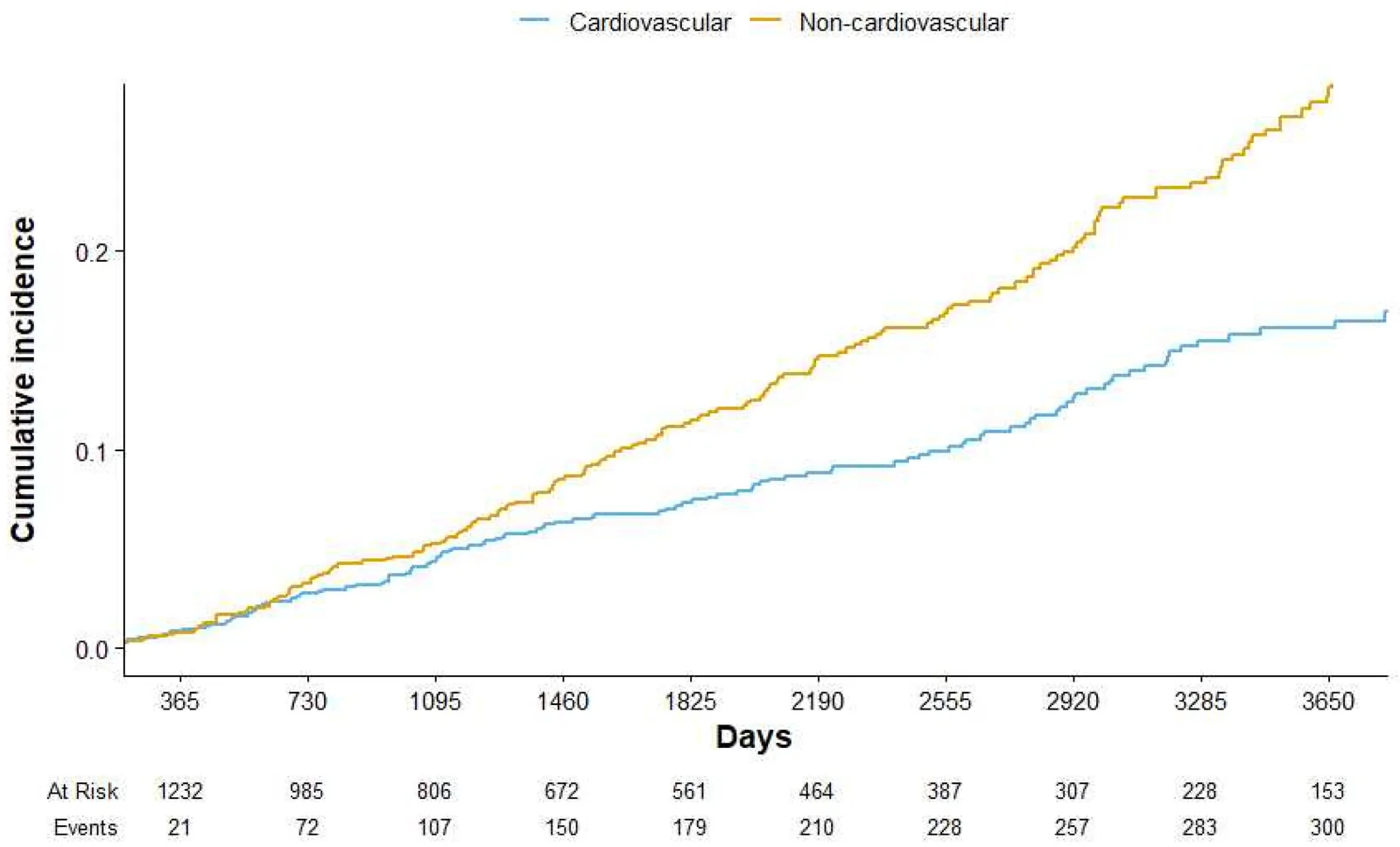
Cumulative incidence of cardiovascular and non-cardiovascular mortality during long-term follow-up (up to 10 years of follow-up). The blue and orange lines represent the cumulative incidence curves of cardiovascular and non-cardiovascular mortality, respectively, in patients who survived the first year following the initial consultation (*n* = 1232) and had data from a follow-up echocardiogram available. The lower section indicates the number of patients at risk and the cumulative number of events in each time interval.

The long-term cumulative incidence curves (Figure 2) reflect this prognostic divergence. After the first year, the trajectory of non-cardiovascular mortality shows a steeper and more constant upward slope compared with cardiovascular mortality. This gap between the two curves widens progressively and steadily as follow-up progresses, consolidating the shift in pattern towards non-cardiovascular processes in the advanced stages of the disease, coinciding with the gradual decline in the remaining at-risk population (from 1232 patients at 365 days to 153 patients at 3650 days, with a cumulative of 300 events recorded at the evaluated time points).

**Figure 3:**
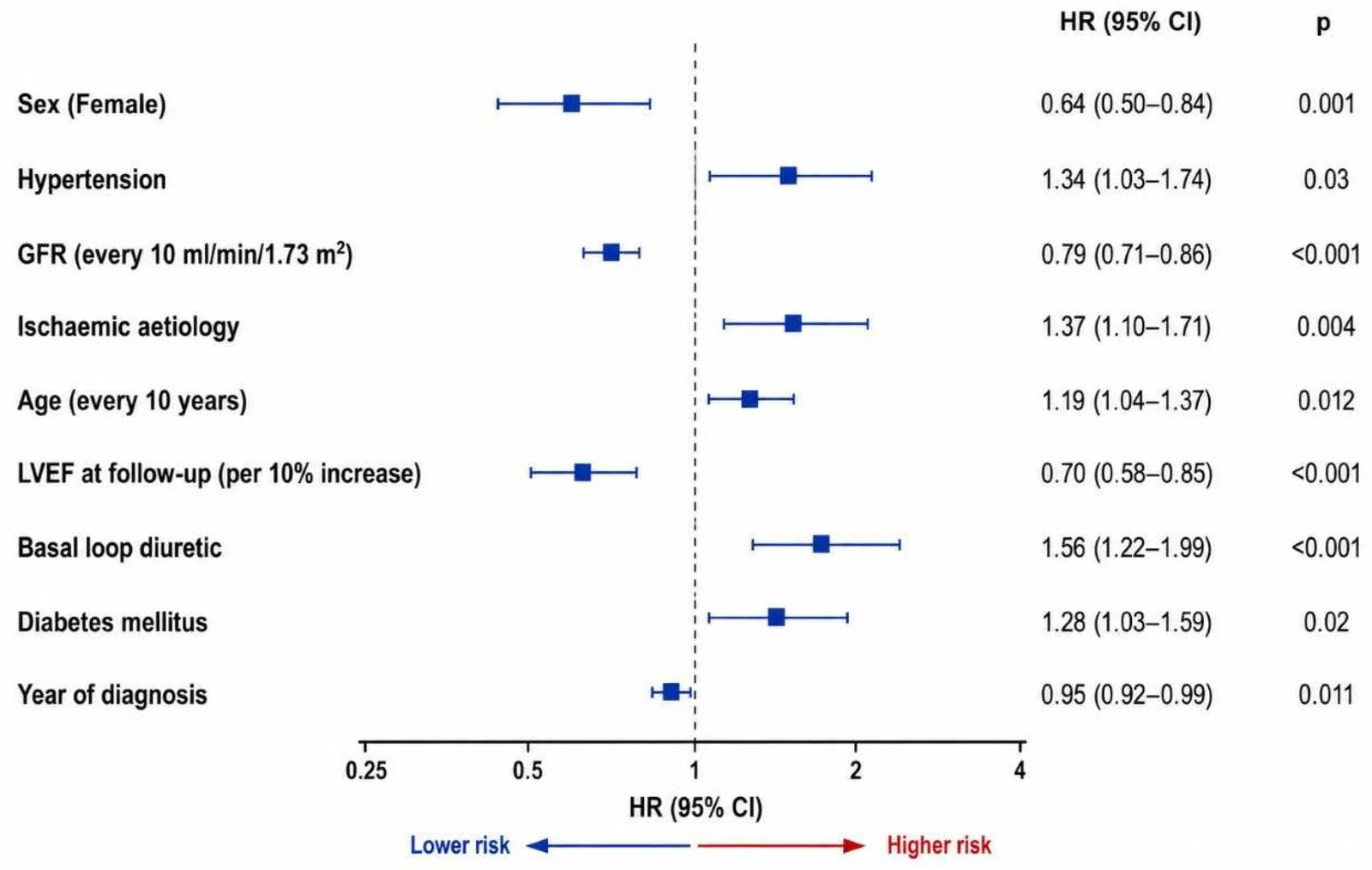
Independent predictors of late all-cause mortality (>1 year of follow-up). Forest plot presenting multivariable Cox proportional hazards regression analysis for all-cause mortality beyond the first year. Point estimates (blue squares) represent hazard ratios (HR) and error bars indicate 95% confidence intervals (CI). The vertical dashed line represents the reference line of no effect (HR = 1.0). Female sex, higher glomerular filtration rate (GFR, per 10 mL/min/1.73 m^2^ increase), follow-up left ventricular ejection fraction (LVEF, per 10% increase), and a more recent year of diagnosis were independently associated with lower mortality. Conversely, baseline loop diuretic use, ischemic etiology, hypertension, diabetes mellitus, and older age (per 10-year increase) independently portended higher mortality risk (*p* < 0.05 for all). CI = confidence interval; eGFR = estimated glomerular filtration rate; HR = hazard ratio; LVEF = left ventricular ejection fraction.

Table 8 and the corresponding forest plot (Figure 3) show the results of the multivariate analysis using Cox regression, which identifies the factors acting as independent predictors of long-term all-cause mortality. Among the 1455 patients who survived at least one year, all variables used in the Cox regression model were available in 1244 patients.

#### 3.4.1. Late Mortality from Cardiovascular Causes

HF remained the leading cause of late cardiovascular death (28.5%), followed by sudden death (10.5%), stroke (1.1%) and acute coronary syndrome (ACS) (0.3%) (Table 6).

With regard to the multivariate Cox analysis (Table 9), the variables independently associated with cardiovascular mortality beyond the first year were ischemic etiology, baseline loop diuretic use, age and LVEF at follow-up. All of these, with the exception of follow-up LVEF (per 10% increase: HR 0.44; 95% CI: 0.31–0.61; *p* < 0.001) and year of diagnosis, were associated with a significant increase in the risk of death, ischemic etiology being the most important factor (HR 2.52; 95% CI: 1.76–3.60; *p* < 0.001), followed by loop diuretic use (HR 2.23; 95% CI: 1.48–3.36; *p* < 0.001). As mentioned, a lower LVEF on the second echocardiogram was associated with higher cardiovascular mortality, thus highlighting the prognostic value of changes in LVEF over the initial diagnostic measurement.

#### 3.4.2. Late Mortality from Non-Cardiovascular Causes

Non-cardiovascular causes accounted for most of the late-stage mortality after the first year (59.7%). Cancer was the most common single cause within this group, accounting for 24.3% of all late-stage deaths. Other significant causes included infections or sepsis (7.5%), severe functional decline or frailty (1.9%), unknown causes (11%) and other causes (14.9%, comprising mainly suicides or surgical complications).

Multivariate analysis indicates a distinct risk profile for this type of mortality, linked primarily to comorbidities and baseline status (Table 10). Thus, the variables that increase the risk of late non-cardiovascular death are hypertension (HR 1.58; 95% CI: 1.109–2.20; *p* = 0.013) and diabetes mellitus (HR 1.38; 95% CI: 1.04–1.84; *p* = 0.036). Similarly, a higher glomerular filtration rate (eGFR) (every 20 mL/min/1.73 m^2^; HR 0.74; 95% CI: 0.67–0.82; *p* < 0.001) and being diagnosed in later years (HR: 0.93 95% CI: 0.89–0.98, *p* = 0.006) were associated with a lower risk of late non-cardiovascular mortality, suggesting that poorer renal function identifies patients with greater long-term vulnerability.

The proportional hazards assumption was assessed for all final Cox models using scaled Schoenfeld residuals. No evidence of a global violation was observed in any model (all global *p* > 0.05). A borderline time-dependent effect was detected only for age in the late all-cause and cardiovascular mortality models (*p* = 0.046 and *p* = 0.047, respectively); all other covariates satisfied the proportional hazards assumption.

## 4. Discussion

This study analyses the clinical course of a contemporary cohort of 1510 patients with HFrEF, revealing that mortality is not a static process, but a dynamic one with determinants that evolve according to the stage of the disease. These findings are consistent with large contemporary registries, such as the ESC-HF-LT, which show high 1-year mortality and a significant proportion of deaths of cardiovascular origin [20]. Furthermore, population-based studies have demonstrated that, despite therapeutic advances, HF continues to be associated with high mortality over time [21,22]. The main finding is the transition from an early phase, dominated by renal function and ischemic etiology, to a late phase characterized by multimorbidity and the recovery capacity of the LVEF. These findings challenge the traditional static approach to risk stratification and support the need for a dynamic, stage-adapted prognostic framework in HFrEF.

During the first year following initial contact with the specialist HF unit, cardiovascular and non-cardiovascular mortality showed a nearly balanced distribution (47.2% vs. 52.8%, respectively), with a slight predominance of non-cardiovascular causes. Nevertheless, progression of HF remained the leading individual cause of death during this vulnerable phase. This finding suggests that, even in early stages, multimorbidity and systemic comorbidities already exert a substantial influence on prognosis. This pattern is partially consistent with European registries showing a substantial burden of cardiovascular mortality during the early phases of HFrEF [20].

This initial period represents a phase of high clinical vulnerability, in which baseline severity and ventricular dysfunction decisively determine the prognosis. In this context, preserved renal function was independently associated with lower early mortality, reinforcing the prognostic relevance of renal status during this vulnerable phase.

The close relationship observed between baseline renal dysfunction and early mortality highlights the fundamental role of cardiorenal syndrome as the main obstacle to the optimization of guideline-directed medical therapy (GDMT). In the early stages, the deterioration in eGFR and the need for high doses of loop diuretics (the latter associated with a threefold increased risk of early cardiovascular death) define a cardiorenal phenotype characterized by congestion and clinical instability, which often hinders the adjustment of essential therapies such as ARBs and MRAs. This phenomenon creates a therapeutic barrier in which clinical instability and low renal reserve prevent the benefits of the four pillars of treatment from being realized [23], which is consistent with the clinical inertia described in real-world studies such as TriNetX, where only a minority of patients achieve full optimal therapy, thereby determining the prognosis during the patient’s most vulnerable phase [14]. Taken together, the independent association between ischemic etiology, eGFR and NYHA functional class with early death suggests that, in the early stages of the disease, the prognosis is closely linked to clinical severity and the patient’s baseline characteristics [22,23]. This is consistent with large observational registries, where renal dysfunction, functional class and age have been shown to be robust predictors of mortality [24,25].

From the first year onwards, a paradigm shift can be observed, with non-cardiovascular causes becoming predominant, accounting for 59.7% of deaths. This shift towards a greater prominence of non-cardiovascular causes is consistent with recent epidemiological data showing a progressive increase in non-cardiovascular mortality in patients with HF, particularly in settings with a higher burden of comorbidity [9,25]. Whilst death due to progression of HF decreases proportionally, conditions such as cancer and infections take on renewed prominence [21]. This shift in the risk profile, in which HFrEF behaves less as an isolated cardiac condition and more as a systemic syndrome, is reflected in the multivariate models, in which hypertension and diabetes mellitus emerge as the main determinants of late mortality from non-cardiovascular causes. In multivariable analysis, higher baseline eGFR (per $20\mL/min/1.73 m^2^ increase: HR 0.74; 95% CI: 0.67–0.82; *p* < 0.001) and a more recent year of diagnosis (HR 0.93; 95% CI:0.89–0.98; *p* = 0.006) were independently associated with a lower risk of late non-cardiovascular mortality, corresponding to risk reductions of 26% and 7%, respectively. These findings are consistent with the view of advanced HF as a systemic disease, in which chronic inflammation determines the final outcome beyond HF itself.

A key finding of this study is that the follow-up LVEF is a better prognostic indicator than the initial diagnostic measurement when predicting long-term mortality. Although LVEF at diagnosis did not significantly differentiate outcomes, changes during follow-up were independently associated with long-term mortality. In fact, for every 10% increase in LVEF during follow-up, the risk of cardiovascular death is reduced by 56% (HR 0.44; *p* < 0.001). This observation suggests that LVEF during follow-up reflects the dynamic response to treatment and the degree of reverse ventricular remodeling, providing a more accurate reflection of the underlying disease trajectory. In this regard, recovery of LVEF should not be interpreted merely as a functional parameter, but as a biomarker of treatment response and myocardial reserve, with direct implications for risk re-stratification [11,18]. In addition, this observation aligns with the growing body of evidence supporting the concept of HF with improved ejection fraction as a distinct clinical phenotype. Recent systematic reviews and meta-analyses have consistently shown that patients who achieve meaningful improvement in LVEF experience substantially lower mortality and hospitalization rates compared with those who remain within the HFrEF category [26,27]. Several interrelated factors contributed to the LVEF improvement observed in our cohort. Foremost among these was the systematic optimization of GDMT, where neurohormonal blockade and SGLT2 inhibition acted synergistically to reduce ventricular wall stress and facilitate reverse myocardial remodeling. Furthermore, non-ischemic etiologies—such as tachycardia-induced or hypertensive cardiomyopathy—demonstrated a greater potential for LVEF recovery compared to ischemic cases, where permanent myocardial scarring limits functional regain.

Consequently, while baseline LVEF remains essential for initial diagnostic categorization, interval changes in LVEF during follow-up offer a vastly superior prognostic indicator for long-term survival. These findings strongly reinforce current clinical practice guidelines advocating routine repeat echocardiographic assessment following GDMT optimization. Rather than relying on a static, single-point baseline measurement, dynamic and evolving risk stratification models that incorporate individual ventricular response should be adopted to guide long-term clinical decision-making.

From a clinical perspective, these findings support a clinical management approach tailored to the stage of the disease. On the one hand, in the early phase, efforts should focus on the rapid initiation and optimization of GDMT, paying particular attention to renal function as a modifiable limiting factor. Strategies aimed at overcoming treatment inertia and safely optimizing treatment in patients with renal impairment may have a critical impact on early survival. However, the focus in the late phase should shift towards the comprehensive management of comorbidities and the prevention of non-cardiovascular events, recognizing that clinical stability may be compromised by systemic frailty. This shift reflects the need to address the broader systemic determinants of prognosis once the window of greatest cardiovascular vulnerability has passed.

## 5. Conclusions

Mortality in patients with HFrEF is a dynamic process defined by a shift in the determinants of survival depending on the stage of the disease. During the first year, cardiovascular and non-cardiovascular mortality showed a relatively balanced distribution, although HF progression remained the leading individual cause of death. After the first year, the risk profile shifted more clearly towards non-cardiovascular causes, such as neoplasms and infections, driven by biological aging and systemic comorbidities.

These findings advocate for a paradigm shift toward dynamic, stage-adapted prognostic stratification in HFrEF. Clinical management must evolve from early, aggressive guideline-directed medical therapy titration and cardiorenal protection to a long-term, multidisciplinary approach focused on comprehensive comorbidity management.

## 6. Limitations

This study has several limitations that warrant consideration. First, its retrospective, observational, single-center design may limit the generalizability of our findings to other clinical settings. Data extraction from electronic health records carries an inherent risk of reporting bias and unmeasured confounding. Second, cause-of-death adjudication—particularly for non-cardiovascular, unknown, or other non-cardiac events—relied on clinical record documentation, which may be subject to misclassification.

Third, key clinical variables were assessed statically at baseline, including renal function, atrial fibrillation (captured as a binary variable without pattern subclassification), and respiratory comorbidities (recorded without functional staging such as GOLD criteria or apnea–hypopnea index). Consequently, longitudinal cardiorenal dynamics, pattern-specific arrhythmia effects, and the prognostic impact of advanced lung disease could not be evaluated. Furthermore, echocardiographic parameters of right ventricular function and pulmonary hypertension were not systematically available across the entire cohort, precluding their integration into the multivariable survival models.

Fourth, while patient management adhered to ESC guidelines, specific data on device therapies (e.g., ICDs, CRT, or ventricular assist devices) were not incorporated into survival analyses, and unmeasured confounding on sudden death cannot be entirely excluded. Fifth, survival analyses incorporating follow-up LVEF were conditional on surviving to the repeat echocardiographic examination and limited to patients with available follow-up data. Finally, subgroup analyses by age, sex, etiology, or baseline NYHA class were beyond the scope of this study. Given the observational nature of the design, multivariable associations demonstrate prognostic predictability rather than direct causality. Although parsimonious multivariable models were used to reduce model complexity, the limited number of outcome events, particularly in the early mortality and cause-specific analyses, may have increased the risk of overfitting and reduced the stability of the estimated hazard ratios. Therefore, these findings should be interpreted with caution and warrant confirmation in larger cohorts.

## Figures and Tables

**Table 1:** Baseline characteristics.

*p* All Groups	*p* Death Groups	Death After 1 Year (*n* = 362)	Death Within 1st Year (*n* = 55)	Alive (*n* = 1093)	*N*	Total (*n* = 1510)	
*0.04*	0.37	295 (81.5)	42 (76.4)	821 (75.1)	1510	1158 (76.7)	Male sex
*<0.001*	0.77	70.4 ± 8.9	70.7 ± 7.5	64.2 ± 11.2	1509	65.9 ± 10.9	Age, years
0.26	0.24	28.7 ± 4.9	27.9 ± 4.7	29.3 ± 8.6	1456	29.1 ± 7.8	BMI, kg/m^2^
*<0.001*	0.12	65.4 ± 27.6	59.2 ± 25.1	82.5 ± 34.6	1452	77.5 ± 33.7	eGFR, ml/min/1.73 m^2^
NYHA							
*<0.001*	*<0.001*	95 (26.2)	7 (12.7)	358 (33)	1501	460 (30.6)	I
		235 (64.9)	34 (61.8)	674 (62.2)		943 (62.8)	II
		32 (8.8)	14 (25.5)	52 (4.8)		98 (6.5)	III or IV
CKD Stage							
<0.001	0.28	62 (17.7)	7 (13.2)	395 (37.7)	1452	464 (32)	I
		127 (36.2)	18 (34)	366 (34.9)		511 (35.2)	II
		145 (41.3)	22 (41.5)	252 (24.0)		419 (28.9)	IIIa
		16 (4.6)	5 (9.4)	28 (2.7)		49 (3.4)	IIIb
		1 (0.3)	1 (1.9)	7 (0.7)		9 (0.6)	IV
Heart failure primary etiology							
0.004	0.41	155 (42.8)	31 (56.4)	378 (34.6)	1510	564 (37.4)	Ischemic
		53 (14.6)	7 (12.7)	248 (22.7)		308 (20.4)	Idiopathic
		28 (7.7)	4 (7.3)	130 (11.9)		162 (10.7)	Arrhythmic
		33 (9.1)	6 (10.9)	98 (9.0)		137 (9.1)	Enolic
		41 (11.3)	3 (5.5)	88 (8.1)		132 (8.7)	Hypertensive
		52 (14.4)	4 (7.3)	151 (13.8)		207 (13.78)	Other
0.36	0.48	30.2 ± 6.5	30.9 ± 7.2	30.8 ± 6.9	1510	30.7 ± 6.5	LVEF at diagnosis, %
*<0.001*		39.9 ± 12.0	-	44.4 ± 10.7	1329	43.1 ± 12.0	LVEF after 2nd echocardiography
Cardiovascular risk factors							
*<0.001*	0.67	284 (78.5)	41 (75.9)	682 (63.4)	1491	1007 (67.5)	Hypertension
*<0.001*	0.46	161 (44.5)	29 (52.7)	376 (35.3)	1481	566 (38.2)	Diabetes
0.19	0.08	249 (68.8)	30 (56.6)	707 (66.1)	1484	986 (66.4)	Dyslipidemia
0.09	0.83	28 (7.8)	3 (5.6)	105 (9.8)	1491	136 (9.1)	Active smoker
Comorbidities							
*0.01*	0.73	*28 (7.7)*	5 (9.1)	145 (13.3)	1510	178 (11.8)	Respiratory
*<0.001*	0.77	131 (36.2)	21 (38.2)	159 (14.5)	1510	311 (20.6)	Cancer
0.54	0.43	4 (1.1)	-	7 (0.6)	1510	11 (0.7)	Cognitive decline
0.23	0.61	34 (9.4)	4 (7.3)	73 (6.7)	1510	111 (7.4)	Hepatic disease
0.57	0.29	7 (1.9)	-	22 (2.0)	1510	29 (1.9)	Autoimmune disease
0.008	0.96	130 (37.7)	19 (38.0)	281 (29.1)	1360	430 (31.6)	Atrial fibrillation

Data are expressed as frequency (percentage) or mean ± standard deviation. BMI: body mass index; eGFR: estimated glomerular filtration rate; LVEF: left ventricular ejection fraction. N indicates patients with available data.

**Table 2:** Baseline cardiological medical treatment.

*p* All Groups	*p* Death Groups	Death After 1 Year (*n* = 362)	Death Within 1st Year (*n* = 55)	Alive (*n* = 1093)	Total (*n* = 1510)	
0.52	0.62	353 (97.5)	53 (96.4)	1052 (96.2)	1458 (96.6)	Betablockers
***0.03***	***0.04***	350 (96.7)	50 (90.9)	1063 (97.3)	1463 (96.9)	RAASIs (ACEi, ARNI or ARB)
***<0.001***	0.89	194 (53.6)	30 (54.5)	706 (64.6)	930 (61.6)	MRA
***<0.001***	0.92	259 (71.5)	39 (70.9)	549 (50.2)	847 (56.1)	Loop diuretics
***<0.001***	***<0.001***	10 (2.8)	11 (20.0)	398 (36.4)	419 (27.7)	SGLT2i
***<0.001***	0.79	138 (38.1)	22 (40)	649 (59.4)	809 (53.6)	Statins
0.13	0.23	140 (38.7)	26 (47.3)	387 (35.4)	553 (36.6)	Anticoagulants

Data presented as frequency (percentage). RAASIs: renin–angiotensin–aldosterone system inhibitors; ACEi: angiotensin-converting enzyme inhibitors; ARB: angiotensin II receptor blocker; ARNI: angiotensin receptor–neprilysin inhibitor; MRA: mineralocorticoid receptor antagonists; SGLT2i: sodium–glucose cotransporter 2 inhibitors.

**Table 3:** Cause of mortality at 1 year (*n* = 55).

Cardiovascular Death = 26 (47.2)	
4 (7.3)	Sudden death
21 (38.2)	Heart failure
1 (1.8)	Stroke
**Non-Cardiovascular Death = 29 (52.8)**	
16 (29.1)	Cancer
4 (7.3)	Infection/Sepsis
5 (9.1)	Unknown
4 (7.3)	Others

Data presented as frequency (percentage).

**Table 4:** Univariable and parsimonious multivariable Cox regression analyses of predictors of 1-year all-cause mortality.

Univariate Analyses		Parsimonious Multivariate Analyses		
*p*	HR (95% CI)	*p*	HR (95% CI)	
***0.001***	1.60 (1.20–2.13)	***-***	-	Age every 10 years
***0.024***	1.85 (1.08–3.15)	***0.038***	1.78 (1.03–3.08)	Ischemic etiology
***<0.001***	0.65 (0.53–0.80)	***0.001***	0.71 (0.58–0.87)	eGFR, (every 10 mL/min/1.73 m^2^)
***<0.001***	3.41 (2.12–5.50)	***<0.001***	3.38 (2.07–5.52)	NYHA (I, II, III/IV)
***0.03***	1.92 (1.07–3.43)	-	-	Loop diuretic
0.98	1.00 (0.54–1.87)	***-***	-	Sex
0.78	1.01 (0.71–1.55)	***-***	-	LVEF at diagnosis (per 10% increase)
***0.03***	1.77 (1.05–2.99)	***-***	-	Diabetes mellitus
0.17	1.54 (0.82–2.86)	***-***	-	Hypertension
0.07	0.94 (0.87–1.00)	***-***	-	Year of diagnosis

eGFR: glomerular filtration rate; NYHA: New York Heart Association. All prespecified candidate predictors were evaluated in univariable Cox regression analyses. Variables with *p* < 0.05 were entered into the initial multivariable model. Variables were then removed sequentially in a manual stepwise manner until the final parsimonious model was obtained. The initial multivariable model included 6 regression parameters and was based on 53 events; the final model retained 3 regression parameters.

**Table 5:** Univariate and parsimonious multivariate analysis (Cox regression) of cardiovascular death at 1st year.

Parsimonious Multivariate Analyses		Univariate Analyses		
HR (95% CI)	*p*	HR (95% CI)	*p*	
-	-	2.21 (1.38–3.56)	***<0.001***	Age, every 10 years
2.78 (1.20–6.40)	***0.01***	3.23 (1.40–7.4)	***0.006***	Ischemic etiology
0.60 (0.44–0.84)	***0.002***	0.55 (0.40–0.76)	***<0.001***	eGFR (every 10 mL/min/1.73 m^2^)
4.38 (2.18–8.81)	***<0.001***	5.41 (2.71–10.8)	***<0.001***	NYHA (I, II, III/IV)
-	-	4.3 (1.5–12.5)	***0.007***	Loop diuretic
-	-	0.60 (0.20–1.72)	0.33	Sex Female
-	-	0.89 (0.52–1.54)	0.68	LVEF at diagnosis (per 10% increase)
-	***-***	1.60 (0.74–3.4)	0.23	Diabetes mellitus
-	***-***	1.04 (0.44–2.4)	0.93	Hypertension
-	***-***	0.90 (0.81–1.00)	0.05	Year of diagnosis

eGFR: glomerular filtration rate; NYHA: *New York Heart Association*. All prespecified candidate predictors were evaluated in univariable Cox regression analyses. Variables with *p* < 0.05 were entered into the initial multivariable model. Variables were then removed sequentially in a manual stepwise manner until the final parsimonious model was obtained. The initial multivariable model included 5 regression parameters and was based on 26 events; the final model retained 3 regression parameters.

**Table 6:** Univariate and parsimonious multivariate analysis (Cox regression) of non-cardiovascular death at 1st year.

Univariate Analyses		Parsimonious Multivariate Analyses		
HR (95% CI)	*p*	HR (95% CI)	*p*	
1.27 (0.89–1.82)	0.18	-	-	Age, every 10 years
1.16 (0.56–2.41)	0.68	-	-	Ischemic etiology
0.76 (0.58–0.99)	***0.04***	-	-	eGFR (every 10 mL/min/1.73 m^2^)
2.25 (1.16–4.3)	***0.02***	2.25 (1.16–4.3)	0.02	NYHA (I, II, III/IV)
1.11 (0.53–2.33)	0.77	-	-	Loop diuretic
1.46 (0.67–3.21)	0.34	-	-	Sex Female
1.24 (0.71–2.17)	0.45	-	-	LVEF at diagnosis (per 10% increase)
1.95 (0.95–4.02)	0.07	-	-	Diabetes mellitus
2.33 (0.89–6.12)	0.08	-	-	Hypertension
0.97 (0.88–1.06)	0.51			Year of diagnosis

eGFR: estimated glomerular filtration rate; NYHA: *New York Heart Association*. All prespecified candidate predictors were evaluated in univariable Cox regression analyses. Variables with *p* < 0.05 were entered into the initial multivariable model. Variables were then removed sequentially in a manual stepwise manner until the final parsimonious model was obtained. The initial multivariable model included 2 regression parameters and was based on 27 events; the final model retained 1 regression parameter.

**Table 7:** Cause of mortality after 1 year (*n* = 362).

Cardiovascular Death = 146 (40.3)	
38 (10.5)	Sudden death
103 (28.5)	HF
1 (0.3)	ACS
4 (1.1)	Stroke
**Non-Cardiovascular Death = 216 (59.7)**	
88 (24.3)	Cancer
54 (14.9)	Others
27 (7.5)	Infection/sepsis
7 (1.9)	Deterioration
40 (11)	Unknown

Data presented as frequency (percentage). HF: heart failure, ACS: acute coronary syndrome.

**Table 8:** Univariate and parsimonious multivariate analysis (Cox regression) of death after 1st year.

Parsimonious Multivariate Analysis		Univariate Analyses		
HR (95% CI)	*p*	HR (95% CI)	*p*	
1.37 (1.10–1.71)	***0.004***	1.47 (1.20–1.81)	***<0.001***	Ischemic etiology
0.79 (0.71–0.86)	***<0.001***	0.72 (0.67–0.78)	***<0.001***	eGFR (every 10 mL/min/1.73 m^2^)
1.19 (1.04–1.37)	***0.012***	1.55 (1.38–1.74)	***<0.001***	Age (every 10 years)
0.64 (0.50–0.84)	***0.001***	0.73 (0.55–0.95)	***0.02***	Sex Female
1.28 (1.03–1.59)	***0.02***	1.55 (1.25–1.91)	***<0.001***	Diabetes mellitus
1.34 (1.03–1.74)	***0.03***	1.81 (1.38–2.35)	***<0.001***	Hypertension
-	**-**	0.87 (0.74–1.01)	**0.07**	LVEF at diagnosis (per 10% increase)
0.70 (0.58–0.85)	***<0.001***	0.56 (0.46–0.68)	***<0.001***	LVEF at follow-up (per 10% increase)
1.56 (1.22–1.99)	***<0.001***	2.02 (1.59–2.56)	***<0.001***	Baseline loop diuretic
-	**-**	1.41 (1.17–1.70)	***<0.001***	NYHA (I, II, III/IV)
0.95 (0.92–0.99)	***0.011***	0.91 (0.87–0.95)	***<0.001***	Year of diagnosis

eGFR: estimated glomerular filtration rate; LVEF: left ventricular ejection fraction. Survival time was calculated from the date of the follow-up Echocardiography to death or censoring. All prespecified candidate predictors were evaluated in univariable Cox regression analyses. Variables with *p* < 0.05 were entered into the initial multivariable model. Variables were then removed sequentially in a manual stepwise manner until the final parsimonious model was obtained. The initial multivariable model included 10 regression parameters and was based on 358 events; the final model retained 9 regression parameters.

**Table 9:** Multivariate analysis (Cox regression) of cardiovascular death after 1st year.

Parsimonious Multivariate Analyses		Univariate Analyses		
HR (95% CI)	*p*	HR (95% CI)	*p*	
-	-	0.64 (0.41–1.01)	*0.06*	Sex female
2.52 (1.76–3.61)	***<0.001***	2.68 (1.88–3.81)	***<0.001***	Ischemic etiology
1.57 (1.29–1.92)	***<0.001***	1.70 (1.41–2.05)	***<0.001***	Age (every 10 years)
2.23 (1.48–3.36)	***<0.001***	2.55 (1.71–3.79)	***<0.001***	Baseline loop diuretic
-	**-**	0.98 (0.76–1.26)	**0.90**	LVEF at diagnosis (per 10% increase)
0.44 (0.31–0.61)	***<0.001***	0.34 (0.25–0.47)	***<0.001***	LVEF at follow-up (per 10% increase)
-	**-**	1.77 (1.26–2.48)	***<0.001***	Diabetes mellitus
-	**-**	1.95 (1.26–3.0)	***0.003***	Hypertension
-	**-**	0.71 (0.63–0.81)	***<0.001***	eGFR (every 10 mL/min/1.73 m^2^)
-	**-**	1.62 (1.20–2.18)	***0.001***	NYHA (I, II, III/IV)
0.93 (0.87–0.99)	***0.04***	0.88 (0.83–0.95)	***<0.001***	Year of diagnosis

eGFR: estimated glomerular filtration rate; LVEF: left ventricular ejection fraction. Survival time was calculated from the date of the follow-up Echocardiography to death or censoring. All prespecified candidate predictors were evaluated in univariable Cox regression analyses. Variables with *p* < 0.05 were entered into the initial multivariable model. Variables were then removed sequentially in a manual stepwise manner until the final parsimonious model was obtained. The initial multivariable model included 9 regression parameters and was based on 126 events; the final model retained 5 regression parameters.

**Table 10:** Multivariate analysis (Cox regression) of non-cardiovascular death after 1st year.

Parsimonious Multivariate Analyses		Univariate Analyses		
HR (95% CI)	*p*	HR (95% CI)	*p*	
-	-	0.78 (0.55–1.01)	0.78	Sex female
-	-	1.46 (1.27–1.70)	*<0.001*	Age (every 10 years)
1.55 (1.09–2.20)	*0.013*	1.73 (1.24–2.44)	*0.001*	Hypertension
0.74 (0.67–0.82)	*<0.001*	0.73 (0.66–0.80)	*<0.001*	eGFR (every 10 mL/min/1.73 m^2^)
-	-	1.76 (1.30–2.37)	*<0.001*	Baseline loop diuretic
1.38 (1.04–1.84)	*0.024*	1.41 (1.07–1.87)	*0.015*	Diabetes mellitus
-		1.28 (1.01–1.64)	*0.04*	NYHA (I, II, III/IV)
-		1.09 (0.82–1.45)	0.54	Ischaemic etiology
-	-	0.80 (0.66–0.97)	*0.03*	LVEF at diagnosis (per 10% increase)
-		0.77 (0.61–0.99)	*0.04*	LVEF at follow-up (per 10% increase)
0.93 (0.89–0.98)	*0.006*	0.93 (0.88–0.98)	*0.003*	Year of diagnosis

eGFR: estimated glomerular filtration rate; LVEF: left ventricular ejection fraction. Survival time was calculated from the date of the follow-up Echocardiography to death or censoring. All prespecified candidate predictors were evaluated in univariable Cox regression analyses. Variables with *p* < 0.05 were entered into the initial multivariable model. Variables were then removed sequentially in a manual stepwise manner until the final parsimonious model was obtained. The initial multivariable model included 9 regression parameters and was based on 194 events; the final model retained 4 regression parameters.

## Data Availability

The original contributions presented in this study are included in the article. Further inquiries can be directed to the corresponding authors.
